# A Novel Approach to Enhance Mechanical and Thermal Properties of SLA 3D Printed Structure by Incorporation of Metal–Metal Oxide Nanoparticles

**DOI:** 10.3390/nano10020217

**Published:** 2020-01-27

**Authors:** Suhail Mubarak, Duraisami Dhamodharan, Manoj B. Kale, Nidhin Divakaran, T. Senthil, Sathiyanathan P., Lixin Wu, Jianlei Wang

**Affiliations:** 1 CAS Key Laboratory of Design and Assembly of Functional Nanostructure, and Fujian Key Laboratory of Nanomaterials, Fujian Institute of Research on the Structure of Matter, Chinese Academy of Sciences, Fuzhou 350002, China; suhail@fjirsm.ac.cn (S.M.); duraisamidhamodharan@fjirsm.ac.cn (D.D.); manojkale@fjirsm.ac.cn (M.B.K.); nidhin@fjirsm.ac.cn (N.D.); sat.bni@gmail.com (S.P.); 2 University of Chinese Academy of Sciences, Beijing 100049, China; 3 Advanced Research School for Technology and Product Simulation, Central Institute of Plastics Engineering and Technology, Chennai 600032, India; tsenthilsci@gmail.com; 4 National Engineering Research Center for Optoelectronic Crystalline Materials, Fuzhou 350002, China

**Keywords:** nanocomposites, stereolithography 3D printing, semiconducting nanoparticles, mechanical properties, thermal properties, rheological properties

## Abstract

Silver (Ag) ornamented TiO_2_ semiconducting nanoparticles were synthesized through the sol-gel process to be utilized as nanofillers with photo resin to enhance the mechanical and thermal properties of stereolithography 3D printed objects. The as-prepared Ag-TiO_2_ nanoparticles (Ag-TNP) were typified and qualified by XRD, XPS, Raman, and FESEM; TEM analysis dissected the morphologies. The enhancement in the tensile and flexural strengths of SLR/Ag-TNP nanocomposites was noted as 60.8% and 71.8%, respectively, at the loading content of 1.0% *w*/*w* Ag-TNP within the SLR (stereolithography resin) matrix. Similarly, the thermal conductivity and thermal stability were observed as higher for SLR/Ag-TNP nanocomposites, equated to neat SLR. The nanoindentation investigation shows an excerpt hike in reduced modulus and hardness by the inclusion of Ag-TNP. The resulted thermal analysis discloses that the introduction of Ag-TNP can appreciably augment the glass transition temperature (T*_g_*), and residual char yield of SLR nanocomposites remarkably. Hence, the significant incorporation of as-prepared Ag-TNP can act as effective nanofillers to enhance the thermal and mechanical properties of photo resin.

## 1. Introduction

In recent years, Additive or 3D printing technology were noted as remarkable developing democratization of innovation, which guarantees colossal possibilities and has picked up a ton of interests from diverse fields like biomedical science and tissue engineering [1,2,3], printing electronics [4,5], microfluidics [6], and aerospace composites [7,8], etc. The 3D printing technology lies in the fabrication process, which facilitates the layer by layer structure formation of 3D objects with the assistance of computer-aided design (CAD) data [9]. Rapid prototyping (3D printing), and the complexities facilitated by it, were transfiguring manufacturing, beyond just the geometry of the part, but also the chemistry and microstructure within the part with position-precise properties [10,11]. Among all the 3D printing technologies, the stereolithography (SLA) technique was noted as the first patented and most promoted process. The photopolymerization technique acting as a critical mechanism of stereolithography 3D printing technology demonstrates superior performance in the manufacturing of 3D constructions with accuracy, rapid curing, and very micro nanoscale resolution with the presence of ultraviolet (UV) light [12,13]. In addition, the photopolymerization techniques show excellent striking, as well as modest approaches in the polymer chemistry nowadays [14]. Utilization of solar light as a power source for reactions has made it conceivable to decrease the cost of the manufacturing processes while being eco-friendly [15]. Therefore, the photopolymerization had an exceptional pull around world to the approach of numerous engineering applications such as coatings, printing technologies, dental fillings, and the building 3D structures [16,17,18]. The SLA technique transforms a multifunctional urethane or acrylic-based monomers and oligomers into an interconnected polymer through propagation reaction, which was initiated by free radicals or cations formed by light illumination [19,20,21]. Most monomers and oligomers are not able to generate initiating free radicals upon light exposure, and it was crucial to run by with some initiators that will assist in initiate polymerization, through a photochemical reaction [22,23].

Certainly, rapid curing and praiseworthy spatial resolution were observed as most advantageous to this technique, but flimsiness and exceptionally reduced bearing resistance were caused by the serious downsides from non-uniform polymer structural design and bulky cross-linking density, which limit SLA resin applications in many industries [24,25]. However, impressive accomplishments on the arrangement of SLA resins with superior mechanical strength and thermal stability of the 3D printed parts have been acquired by introducing nanofillers like graphene, CNT and nano-sized metal and inorganic particles to the photo resin matrix [26,27,28,29], with the effect of loaded nanofillers within the resin matrix being dynamic to make an exceptional interface to enhance interlayer connection, which would prime to higher load allocation to the polymer matrix from the nanofillers, therefore giving an improved reinforcement effect [30,31,32]. Metal and metal oxide nanoparticles have delighted significant research attention over the recent years [33,34]. Numerous efforts have strived on the preparation of metal-oxide based nanoparticles and their broad applications indecisive from solar cells, electronics, and biosensors to environmental applications such as water refinement and hazardous waste recycling treatments [35,36,37].

The sustainable, low harmfulness, cheap, high mechanical, and thermal performance of Titanium dioxide nanoparticles (TNP) envisaged as one of the most promising semiconductor photocatalysts for 3D printing applications, wherein it has a lowest electronic bandgap energy of about 3~3.2 eV that helps to stimulate better photocatalysis reactions under UV light radiation [38,39]. In recent years, various nanostructured TiO_2_ materials, such as nanoparticles, nanotubes, and nanowires were synthesized via many proposed approaches such as sol-gel, hydrothermal, and anodic oxidation, etc. [40,41,42,43]. The photocatalytic process of semiconducting nanoparticles, followed by the formation of active radical species, resulted from the reduction of molecular oxygen (O_2_) by photostimulated electron–hole pairs [44,45]. In addition to TNP, Ag decorated TNP have also been performed as free-radical photoinitiators for curing photo resins, due to their low energy bandgap compared to pristine TNP and proficiency of producing electron–hole pairs which was much needed for the generation of reactive species to improve photopolymerization [46,47,48,49]. Many efforts have been made to improve the thermal and mechanical properties of the polymer matrix by the introduction TNP as nanofillers [50,51].

In this work, we emphasize the importance of pioneering coordination of nanotechnology and additive manufacturing technology, which were made a technological revolution in this era to lead immeasurable promises in science and technology. Silver decorated TiO_2_ (Ag-TNP) is known to be an outstanding photoactive compound that is non-hazardous, cheap, and highly stable [52,53,54]. Here, we have reported the utilization of Ag decorated semiconducting TNP (Ag-TNP) as an effective initiator for the photopolymerization of acrylic-urethane resin under UV irradiation. Our research outcome was revealing that the Ag-TNP can initiate the photopolymerization of methyl methacrylate (MMA) and polyurethane in organic systems in the presence of UV light irradiation. It further shows that the Ag-TNP can recruit O_2_ in the polymerization mechanism to avert inhibition of the initiating radicals. In addition, this Ag-TNP is able to separate easily from the polymer mixture and reprocessed without any substantial worsening in the photocatalytic activity. This metal–metal oxide semiconducting nanostructured filler helps to attain great dispersion and allow the UV light to penetrate maximum depth within the resin system remarkably. The as-prepared Ag-TNP incorporated SLA 3D printed structure exposes very high mechanical and thermal properties due to the enhanced filler dispersion within the polymer matrix and induced photopolymerization achieved via the lower bandgap energy of the semiconducting metal oxide nanoparticles.

## 2. Experimental Procedures

### 2.1. Materials

Macklin, City, China was supplied with Titanium tetrachloride (TiCl_4_, GR, 99.5%), the combination of urethane-acrylate based UV curable stereolithography resin which consists of various monomers and oligomers such as CN9010 (Aliphatic urethane acrylate oligomers), (Sartomer America, Exton, PA, USA), CN991 (Aliphatic polyester based urethane diacrylate oligomers), (Sartomer America, USA), SR209 (Tetra ethylene glycol dimethacrylate), (Sartomer America, USA), HEMA (Hydroxy ethyl methacrylate) and HPMA (Hydroxy propyl methacrylate), (Sigma Aldrich, Wuxi, China), TPO (2,4,6-trimethyl benzoyl diphenyl phosphine oxide) (photoinitiator). Silver nitrate (AgNO_3_, 99%,) and Ethanol (C_2_H_6_O, AR, Anhydrous, 99.7%,) were brought from Sigma Aldrich, China. Methyl alcohol (CH_4_O, 99.5%,) was purchased from Shanghai Titan Scientific Co. Ltd., Shanghai, China. The coupling agent KH570 (3-Glycidoxypropyl tri-methoxy silane) was procured from Wenhua Chemicals, China. The ultra-pure (DI) water was utilized throughout the research work.

### 2.2. Methods

#### 2.2.1. Synthesis of Anatase TiO_2_ NPs (TNP) and Ag-TiO_2_ NPs (Ag-TNP)

Anatase TiO_2_ nanoparticles (TNP) were synthesized via the sol-gel method [55]. In addition, 10 mL TiCl_4_ was slowly added dropwise into 100 mL of absolute ethyl alcohol at room temperature. A significant amount of HCl gas was exhausted for the period of the mixing process. A light yellow solution was acquired and accumulated for several days (2 to 5 days) to form a sol-gel TNP. Then, this reaction mixture was dehydrated at 70 °C until a dry powder was achieved. The dry whitish powder was annealed at 400 °C with a heating rate of 5 °C/min for 30 min to obtain TNP. In order to prepare Ag-TNP, 1 g of TNP was added to the solution containing 10 mL of 100 mM AgNO_3_ and 10 mL of methanol, and the reaction mixture was treated under the irradiation of UV light for 1 h. The solution turns from a milky whitish color to brownish color, which shows the successful decoration of Ag particles on the surface of TiO_2_ NPs. Finally, the brownish solution was cleaned by DI water and ethanol followed by dehydration in a hot air over at 80 °C for 12 h to achieve Ag-TNP powder [56]. The supplementary Figure S1 represents a detailed schematic illustration of the synthesis process of Ag-TNP.

#### 2.2.2. Preparation of Nanocomposites for SLA 3D Printing

To prepare the Ag-TNP incorporated SLA resin nanocomposites, a measured amount of Ag-TNP was treated with coupling agent KH570 (1% *w*/*w* based on filler concentration) in ethanol solution, and dried at 80 °C in a blast oven for 2 h, prior to the addition with SLA resin to get a better dispersion of Ag-TNP within the photo resin by the assistance of ultrasonication followed by continuous stirring [57]. The loading ratio of Ag-TNP in the following description of this work was expressed as the weight (g) of added Ag-TNP per 100 g SLR matrix (denoted by % *w*/*w*). The sample named as SLR for the neat SLA photo resin and the samples, expressed by SLR/Ag-TNP-0.5, represents the loading concentration of 0.5 g as-synthesized Ag-TNP with the 100 g of SLA photo resin and the same as for other samples likes SLR/Ag-TNP-0.8, SLR/Ag-TNP-1.0, and SLR/Ag-TNP-1.2. Without specification, the incorporation of Ag-TNP in the SLA was stated as the weight percentage (% *w*/*w*), and the as-geared SLR nanocomposites with *k* % *w*/*w* loading of Ag-TNP were signified by SLR/Ag-TNP-*k*. The step by step detailed preparation route of SLR/Ag-TNP nanocomposites was demonstrated in the supplementary image Figure S2.

#### 2.2.3. Fabrication of Three-Dimensional Structures by SLA 3D Printer

**Figure 1:**
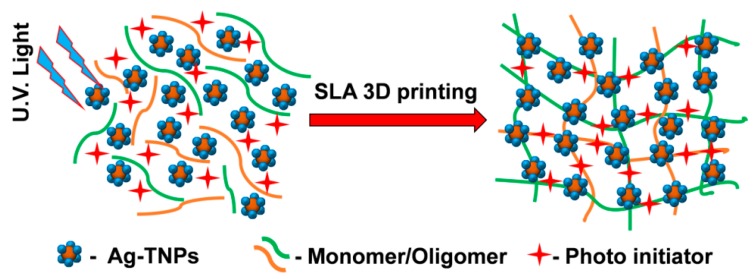
The schematic illustration of the induced photopolymerization of SLR (stereolithography resin) with the incorporated semiconducting Ag-TNP (Ag decorated TiO_2_ nanoparticles) hybrids under UV light for SLA (stereolithography) 3D printing.

Fabrications of neat SLR and SLR/Ag-TNP composite samples were printed with the aid of a Dream 3D-C200 SLA 3D printer with UV laser irradiation power was about 155 mW/cm^2^ and the wavelength of 405 nm followed (refer to supporting information Figure S3) according to the ASTM standard D638. The laser irradiation was about 155 mW/cm^2^ fixed. The scan rate was maintained to 2500 mm/s. After printing, the samples were sluiced by iso-propanol several times and post-cured for 20 min with the assistance of UV chamber. Figure 1 represents the induced photopolymerization reaction by the incorporation Ag-TNP within SLA 3D printing. The optimized monomers and oligomers compositions of prepared SLA resin were presented with the supplementary Table S1.

### 2.3. Measurements and Characterization

The crystalline microstructures phases of as-prepared TiO_2_ and Ag-TNP were determined by low angle XRD (STOE-STADV diffractometer, STOE Corporation, Chicago, IL, USA) using Cu-Kα radiation (λ = 1.59041 Å). A Raman spectrum was recorded with the assistance of a Raman Microscope (Renishaw, Invia, New Mills, UK) with a laser excitation wavelength of about 537 nm. The Raman spectra of TiO_2_ and Ag-TNP powder were observed. The surface composition of elements and electronic states of the Ag-TNP were investigated by using X-ray photon spectroscopy (Thermo Scientific Escalab 250Xi, Waltham, MA, USA). High-resolution transmission electron microscopy (HRTEM) (JEOL JEM-2010, Tokyo, Japan) was engaged to perceive the morphologies of the ultrathin sectioned samples of Ag-TiO_2_NPs and selected area diffraction patterns (SAED) were determined at an acceleration potential was 200 kV. A field emission scanning electron microscope (FESEM) (JSM-7500F, SU8010/EDX, Tokyo, Japan) was intended to observe the morphologies of Ag-TiO_2_NPs, and the fractured surfaces of 3D printed samples after performing the tensile strength analysis. A UV light auxiliary of DHR-2 (Discovery Hybrid Rheometer-2, TA instrument, New Castle, DE, USA) was employed to examine the Rheological study of neat SLR and SLR/Ag-TNP samples. A UV light appurtenant was expended for the dynamics examines of the curing procedure. The series of steady-state shear speed was followed from the range about 0.1 to 100 per seconds, and the temperature was maintained at 25 °C. The upper and lower geometry was prepared by aluminum and a transparent PMMA (Poly methyl methacrylate), respectively, with a diameter about 20 mm for each. The investigation was performed for 80 s in total, and the light source was switched ON at the 30th second with 155 mW/cm^2^ irradiation power being maintained. Thermogravimetric analysis (TGA) was performed by the assistance of TA Instruments STA449C (New Castle, DE, USA) to examine the thermal stability of the 3D printed samples of neat SLR and SLR/Ag-TNP with different concentrations. Each testing was performed about 5–10 mg, and the analyzing temperatures were maintained at starting from 25 °C to 800 °C with an increasing heat rate of 10 °C/min under a nitrogen atmosphere.

Thermo-mechanical analysis or dynamic mechanical analysis (DMA) of neat SLR and SLR/Ag-TNP 3D printed specimens were investigated by the aid of DMA Q800 System (TA Instruments, New Castle, DE, USA) using double cantilever mode. The testing temperature was performed from 25 °C to 180 °C at a heating rate of 5 °C/min at a frequency about 1 Hz. The wavelength of the light absorbance by the materials and its bandgap energy was investigated via the Diffusive Reflectance Spectroscopy (DRS) (PerkinElmer Lambda 950 UV/VIS/NIR spectroscopy, Borken, Germany) instrument. The bandgap energy of as-prepared TiO_2_ and Ag-TiO_2_ was determined by using a mathematical approach of the Kubelka–Munk function. The mechanical properties of neat SLR and SLR/nanofillers 3D printed specimens were analyzed with the help of a universal testing machine, made by AGX-100 plus, Shimadzu, City, Japan. The samples (length 60 mm × thickness 2 mm × gauge distance 45 mm) were tested at a strain rate of 5 mm/min. To a better understanding, the five sets of samples were analyzed for each group of different concentrations. The nanoindentation study was carried out by a nanoindenter apparatus (Hysitron Inc., Tribo Indenter 750, Minneapolis, MN, USA) with an assistance of 3-regions Berkovich diamond indenter and the radius of the indenter probe was fixed at 50 nm. A sample size with 8 × 8 indentations were performed for each sample and the space between two corresponding indentations was set as 10 µm. Thermal conductivity of the as-prepared neat SLR and SLR/nanofillers 3D printed samples were calculated by TC 3000 thermal conductivity tester (XiatechInstrument Factory, Beijing, China) with the aid of a transient hot-wire method at room temperature. The dimensions of each sample length 30 mm × width 10.00 mm were maintained. The photo kinetic study was investigated with the assistance of a real-time FTIR instrument for the neat SLR and SLR/nanofillers, in order to determine the photopolymerization process enhancement by the incorporation of Ag-TiO_2_ nanoparticles. The real-time curing process was carried out for different time interval about 10 s, 30 s, 60 s, 120 s, 240 s, 400 s, and 600 s, respectively. Different power energy was also utilized to analyze the kinetic study of the SLR/nanofillers blends.

## 3. Results and Discussion

### 3.1. Characterization of Ag-TiO_2_ Nanoparticles (Ag-TNP) 

**Figure 2:**
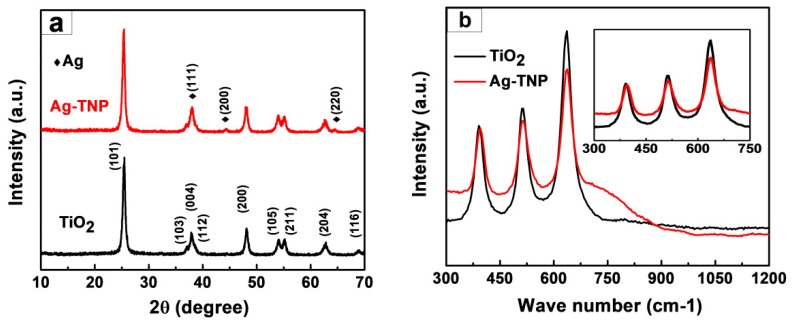
The crystalline structure analysis of as-prepared TiO_2_ and Ag-TNP hybrids (**a**) X-ray diffraction peaks of unmodified TiO_2_ (black pattern), Ag-TNP (red pattern), and (**b**) Raman spectrum of TiO_2_ (black spectrum) and Ag-TNP (red spectrum), inset in (**b**) shows the enlarged view of the Raman spectrum at the wavelength range of 300 to 750.

XRD investigated the crystalline structure and phase of the as-prepared TiO_2_ and Ag-TNP, and the testing was carried out with the range of 2θ lies between 10°–70°. Figure 2a depicts the XRD patterns of TiO_2_ (refer black patterns in Figure 2a) and Ag-TNP (refer red patterns in the Figure 2a), the pristine TiO_2_ exhibited firm diffraction peaks at 25.4°, 37.2°, 37.86°, 38.58°, 48.18°, 53.95°, 55.22°, 62.83°, and 68.91° corresponding to the planar structures of (101), (103), (004), (112), (200), (105), (211), (204), and (116), respectively, and the observed planar was perfectly matched and confirmed with phases of tetragonal anatase TiO_2_ (JCPDS card no: 21-1272). In the case of Ag-TNP, XRD patterns exhibited additional reflections at 2θ values about 38.03°, 44.33°, and 64.48°, which can be attributed to the crystal planes of metallic silver (111), (200), and (200), respectively, and the noted crystalline planar was matched and confirmed with the characteristics of fcc (face-centred cubic) metal (JCPDS card no: 89-3722) nanoparticles about 5 to 10 nm in diameter [58]. The above results were confirming that the Ag particles were successfully decorated on the surfaces of the TiO_2_ NPs.

Raman spectroscopy analysis was performed to investigate the successful ornamentation of Ag NPs on the surfaces of TiO_2_ NPs. Figure 2b demonstrates the Raman spectrum of the TiO_2_ (refer to the black spectrum in Figure 2b) and Ag-TNP (refer to the red spectrum in Figure 2b). The Raman spectrum of TiO_2_ polymorphs was unique enough, and they were beneficial for distinguishing various TiO_2_ phases. The TiO_2_ NPs demonstrated Raman bands observed at 391.2 cm^−1^, 514.6 cm^−1^, and 636.4 cm^−1^ were associated with the B1g, A1g, and Eg Raman modes of anatase TiO_2_, respectively. Conversely, the Ag-TNP Raman spectrum also expresses the Raman bands the same as TiO_2_, but, with the slight deviation, was observed in Eg Raman mode, which was shifted from 636.4 cm^−1^ to 637.5 cm^−1^ (refer to the inset graph in Figure 2b). The observed red-shifting of Eg Raman mode on the Ag-TNP spectrum confirmed that Ag nanoparticles successfully decorated the TiO_2_ surfaces [59].

The X-ray photoelectron spectroscopy (XPS) was a great tool to investigate the composition of elements and electronic states of the samples. The performed XPS analysis graphs were depicted with the supplementary Figure S4. The resulted XPS survey spectrum of pristine TiO_2_ (refer to the black line in Figure S4a) and Ag-TNP (refer to the red line in Figure S4a) were displayed in Figure S4a. In the case of the TiO_2_ survey spectrum, there was no observation of the Ag absolute peak, but the Ti (at 460 eV) and O (at 531 eV) peaks were noted. However, the Ag-TNP survey spectrum exposing the presence of Ti, Ag and O elements was ensured by the intensive peaks around 460 eV (Ti 2P_3/2_ at 459.37 eV and Ti 2P_1/2_ at 465.13 eV), 370 eV (368.1, 374.1), and 531 eV (O-Ti at530.60 eV and O-H at 531.98 eV) binding energy, respectively, in the TiO_2_ samples with its significant contribution. The experiential results of the XPS survey spectrum confirming that the successful adornment of Ag nanoparticles on the surfaces of TiO_2_ NPs. Moreover, the deconvoluted XPS spectra of Ti, Ag, and O were displayed in Figure S4b,c,d, respectively. The Ag 3d_3/2_ and Ag3d_5/2_ peaks were fruitfully indicated in the presence of Ag in Ag-TNP.

**Figure 3:**
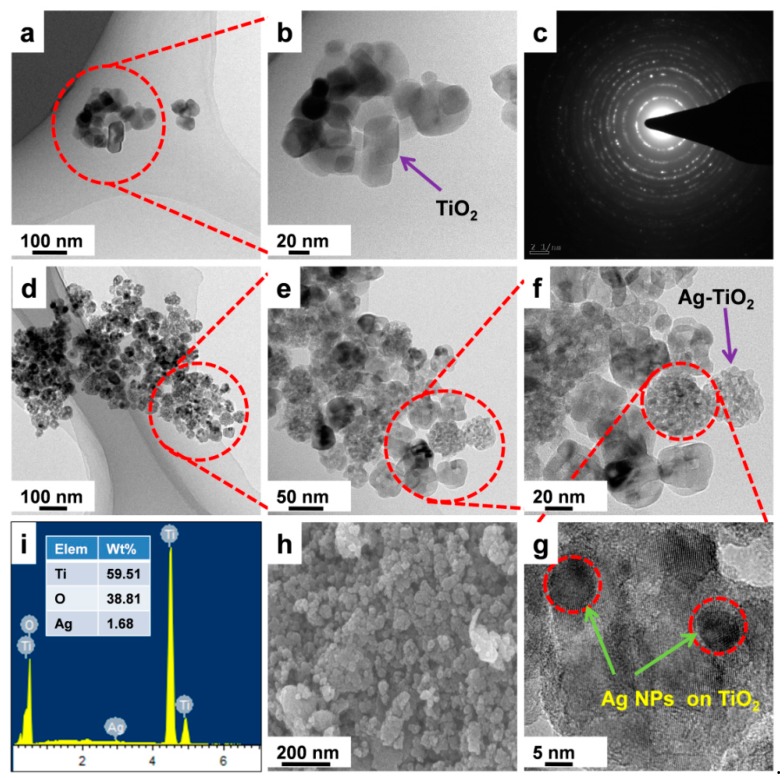
Structural Morphology study analysis of as-prepared TiO_2_ NPs and Ag-TNPs hybrids. (**a**) unmodified TiO_2_, (**b**) neat TiO_2_ at higher magnification about 20 nm, (**c**) the SAED pattern of Ag-TNPs, (**d**) Ag nanoparticles modified TiO_2_, (**e**) and (**f**) higher magnification of Ag-TNPs hybrid, (**g**) a single particle magnification of Ag-TNPs, (**h**) FESEM image of Ag-TNP, (**i**) SEM-EDS report of Ag-TNP.

HRTEM and FESEM images investigated surface morphology and particle size distributions of as-prepared pristine TiO_2_ and Ag-TNP. The samples were dispersed with pure ethanol and drop-casting on a copper grid before analysis. The pristine tetrahedral TiO_2_ NPs were clearly observed with the HRTEM image of Figure 3a,b, with slight agglomeration. However, Figure 3d–f express an experiential HRTEM image of the Ag-TNP with different magnification, which embraced the great agglomeration of approximately tetragonal TiO_2_ and spherical Ag NPs having mean diameters about 30–40 and 5–10 nm, respectively (refer to Figure 3f). For a better understanding, the as-prepared Ag-TNPs were investigated through higher magnification about 5 nm to analyze the single Ag modified TiO_2_ hybrids, and the resulting image was displayed in Figure 3g. Lattice fringes and associated d-spacing (refer to Figure 3c) were consistent with different planes of tetragonal anatase, with the help of the SEM-EDS report confirming that the Ag-TNP were possessed exclusively of Ti, Ag, and O (refer to Figure 3i). The Ag-TNP sample had higher contrast and spherical Ag nanoparticles of 5–10 nm diameters, which appear to embellish on the larger surface of TiO_2_ crystallites uniformly. The analysis of lattice fringes reveals that d-spacing consistent with the planes of the tetragonal structure of anatase TiO_2_ and the planes of spherical Ag NPs, and hence these smaller particles were attributed to metallic Ag nanoparticles. Figure 3c is the SAED pattern of the Ag-TNP generated in HRTEM, which exhibits high crystalline structure and planes. Moreover, the surface morphology study of Ag-TNP was proved that the Ag nanoparticles were successfully accumulated on the TiO_2_ surfaces. In addition to that, Figure 3i expresses the SEM-EDS results of as-prepared Ag-TNP hybrids, and the results revealed that the elemental composition present in the Ag-TNP, while screening that the Ti, O, and Ag elements were only present within the Ag-TNP hybrids, confirmed that the Ag nanoparticles were successfully festooned on the TiO_2_ surfaces.

**Figure 4:**
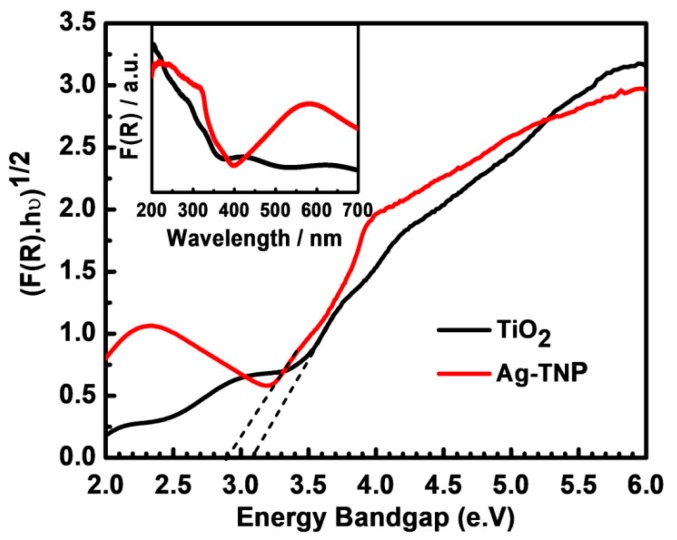
The diffusive reflectance analysis of as-prepared TiO_2_ and Ag modified TiO_2_ hybrids to obtained Tauc’s plot for an indirect bandgap model (Kubelka–Munk function). The red line belongs to Ag-TNP hybrids, and the black line belongs to unmodified TiO_2_ nanoparticles. The dash-dot lines represent the tangent line towards the point, where the line intersects the horizontal energy axis (bandgap). The inset graph shows the enlarged version of the bandgap model study.

The diffusive reflectance spectroscopy (DRS) was an effective analysis to determine the energy bandgap of semiconducting TiO_2,_ and Ag-TNP using the Kubelka–Munk equation, and the calculated results were presented in Figure 4. According to the analysis, the Kubelka–Munk function F(R) allowed the UV absorbance of the semiconducting metal–metal oxide NPs to be estimated from its reflectance (R) according to the following equation:

F(R) = (1 − R)^2^/2R (1)

The bandgap measurement was done by the slope of the Tauc’s curve linearly extended towards the point, where the line intersects the horizontal energy axis employing the subsequent Tauc’s plot appearance when supposing that TiO_2_ was an indirect bandgap semiconductor:

(F(R)·*hv*)^1/2^ = A (*hv* − Eg) (2)

where A, *h*, and *v* were a constant, the Planck constant, and the frequency, respectively [60,61].

From the graph (refer to Figure 4), it was precisely measured that the bandgaps of TiO_2_ and Ag-TNP were 3.1 eV and 2.9 eV, respectively, and also the inset of Figure 4 expressed that the Ag-TNP exhibited a stronger, red-shifted absorbance than TiO_2_ NPs. The lowest energy bandgap would help the semiconducting Ag-TNP undergo rapid and enormous generation of hole–electron pairs under the UV light, which triggers the vast and robust polymerization throughout the photo resin [62].

### 3.2. Characterization of SLR/Ag-TNP Nanocomposites

**Figure 5:**
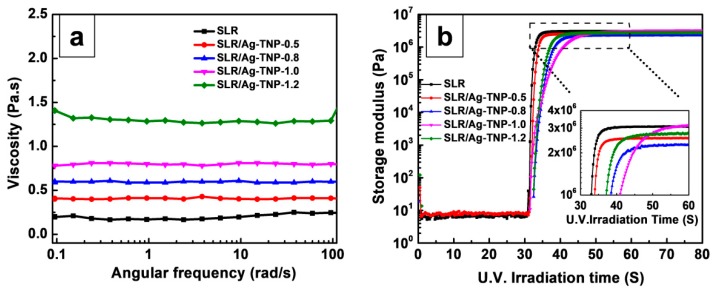
The DHR-2 UV LED analyses of neat SLR and SLR/Ag-TNP nanocomposites (**a**) viscosity variations of SLR/Ag-TNP and neat SLR nanocomposites as a role of shear speed at different concentrations of Ag-TNP loading, (**b**) the consequence of Ag-TNP on the dynamics of the UV light curing response. The mini insert graph in the (**b**) was an engorged sight of the curves among time = 30 to 60 s (a black dotted rectangular line marked the exact focused area).

The UV LED assisting DHR-2 investigation was carried out before the mechanical property analysis of the neat SLR and SLR/Ag-TNP nanocomposites, in order to examine the effects of incorporated Ag-TNP curing behaviors with the SLA resin matrix. The DHR-2 analysis outcome reveals that the SLR/Ag-TNP nanocomposites own an elevated viscosity compared to neat SLR, and the viscosity shows an increase as the loading percentage of Ag-TNP increases from 0.5% to 1.2% *w*/*w* (refer to Figure 5a). Moreover, the DHR-2 operational with a UV LED co-conspirator instrument can be traced as the variations in storage modulus of the nanocomposite resin in the course of the curing route by a fast sampling process. Figure 5b demonstrates the curing behaviors of neat SLR and SLR contains with different loading % *w*/*w* of Ag-TNP. The following protocol was strictly maintained for DHR-2 analysis of tested samples, and the resulting outcomes were experiential in the following trend, which means that, prior to the UV light being switched ON (at time <30 s), the storage modulus of all samples stands for the homogeneous storage modulus, meaning that there were no notable storage modulus changes in the uncured resin. However, once the UV light was switched ON (at time = 30 s), the outstanding increasing trend of storage modulus was renowned, signifying that the cross-linking reaction has proceeded remarkably. After UV curing (at time ≈ 50 s), the cross-linking and curing responses were observed as a saturation level, and the storage modulus was kept in a maximum state and remained steady. The excellent storage modulus was achieved at the loading concentration of 1.0 % *w*/*w* Ag-TNP (refer to the violet line in insert Figure 5b). The resulting graph (refer to Figure 7b) revealed that the UV light may evade the well-dispersed minuscule Ag-TNP very quickly, especially at 1.0 % *w*/*w* of loading concentration with an SLR matrix. Nevertheless, the accumulation of Ag-TNP would obstruct the light and inhibit the photopolymerization in the SLR system [63,64]. Consequently, as the incorporation of Ag-TNP hybrids reaches 1.2% *w*/*w*, significant protracted escalation of polymerization and an apparent decline of curing rate was distinguished. For the UV-instigated photopolymerization and interconnection, the nanoparticles fillers affect the gel time in two contradictory features: (a) the reinforcing effect of nanoparticles may endorse the interconnection between the monomers and oligomers to enhance the modulus of resin matrix, hence reducing the gel period; (b) the nanofillers may obstruct the light, hindering the photopolymerization, and thus extend the gel period [65]. Figure 5b depicts that the accumulation of Ag-TNP lengthens the gel time a little bit, though the highest storage modulus was observed since it was subjected to the strengthening effect (refer to the purple line in Figure 5b).

#### 3.2.1. Mechanical Properties

**Figure 6:**
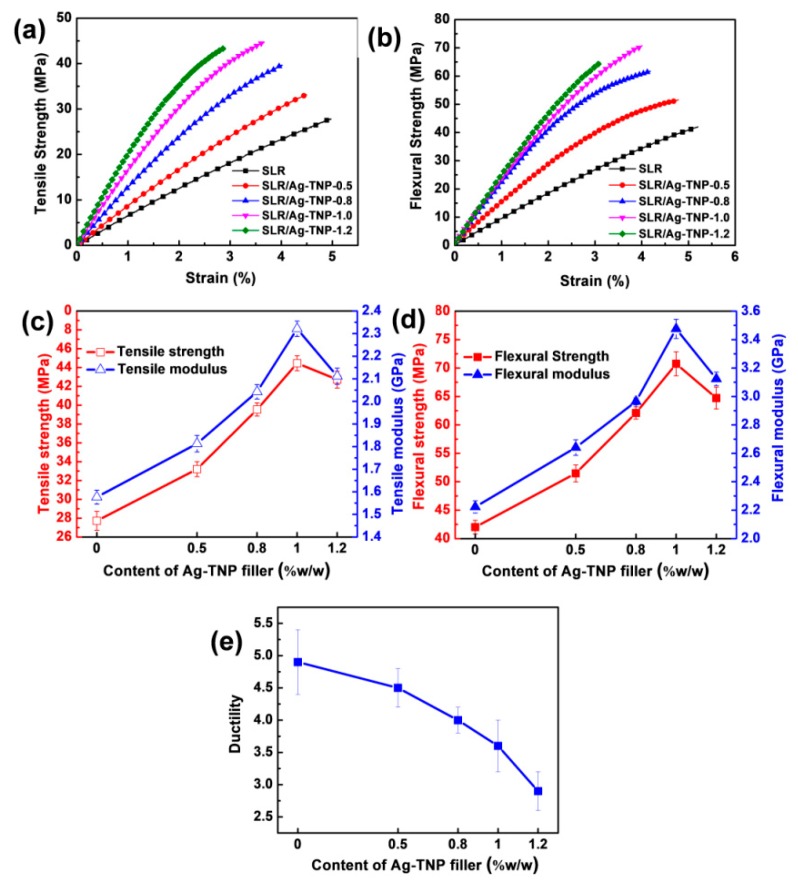
The mechanical properties analysis of neat SLR and SLR/nanofillers. (**a**) the comparative tensile strength analysis of SLR/Ag-TNP at different concentrations, (**b**) the comparative flexural strength analysis of SLR/Ag-TNP at different concentrations, (**c**) the complete data set of tensile study analysis of neat SLR and SLR/Ag-TNP nanocomposites, (**d**) flexural strength and flexural modulus, with SLA 3D printed samples prepared with various concentration SLR/Ag-TNP, and (**e**) the ductility study analysis of SLR/Ag-TNP at different concentrations.

The mechanical behaviors of the SLR/Ag-TNP and neat SLR, performed with the assistance of the universal testing machine and the observed results, were discussed in this subsection. The tensile and flexural stress–strain curves of the stated dog-bone shaped 3D printed samples of neat SLR, and SLR/Ag-TNP nanocomposites were displayed with Figure 6a,b. For a better understanding of the effects of incorporated Ag-TNP with an SLR matrix, mechanical properties enhanced the complete data set of tensile strength and a tensile modulus graph as depicted in Figure 6c,d. The tensile strength of the SLR/Ag-TNP nanocomposites revealed an increasing trend, with increasing loading concentration of Ag-TNP. Especially at the loading content of 1.0% *w*/*w*, Ag-TNP shows remarkable tensile strength enhancement compared with neat SLR. The enhanced tensile strength of SLR/Ag-TNP was noted as 44.7 MPa at the 1.0 % *w*/*w* incorporation of as-prepared Ag-TNP, which shows a 60.8% enhancement compared with neat SLR (27.8 MPa) (refer to the red line in Figure 6c). In addition, the increased tensile modulus was also being observed with the increasing Ag-TNP loading with the SLR matrix (refer to the blue line in Figure 6c). In addition, Figure 6e demonstrates the elongation at break analysis of the neat SLR and SLR/nanofillers nanocomposite, which illustrates that the elongation at break reduces with escalating of the nanofillers (Ag-TNPs) content. The integrated nanofillers may confine the SLR chain segmental mobility to improve the inflexibility of the polymer matrix and make the SLR system more brittle, and then lastly directs to the destitute elongation at break [66]. Concurrently, the ductility of the SLR nanocomposites was abridged while inserting the reinforcing nanofillers into the SLR system, which would appreciably diminish the stress transfer [25,61] and make the SLR system more fragile with inferior ductility caused by the more complicated chain mobility by the rigidity nature of the incorporated nanofillers [62,67]. However, the incorporated nanofillers (Ag-TNPs) render striking enrichment in mechanical properties, owing to their incredible mechanical properties [68,69]. The ductility of 3D printed SLR/Ag-TNP-1.2 (2.9) was reduced 40.8% less than the 3D printed neat SLR (4.9). The increasing mechanical properties were observed with the incorporation of increasing Ag-TNP, but the unexpected decrement was noted when the loading concentration goes beyond 1.0% *w*/*w* of Ag-TNP. This crucial phenomenon was maybe the possible agglomeration of Ag-TNP within the SLR matrix when the incorporation goes to a higher level. However, the better mechanical enhancement was achieved at lower content assimilation of Ag-TNP (1.0% *w*/*w*), due to the homogenous dispersion of incorporated Ag-TNP. Moreover, the low energy bandgap, high crystalline structure, and elevated dispersion of Ag-TNP within the SLR matrix, which helps with the rapid generation of electron–hole pairs and could enhance photopolymerization by a strong bonding with the monomers and oligomers present in the urethane–acrylate photo resin. Hence, the as-prepared Ag-TNP being able to induce the catalytic activity of photopolymerization along with the nanoscale-sized Ag-TNP that can achieve excellent dispersion within the SLR matrix were the key reasons for the mechanical properties enhancement.

The flexural strength and flexural modulus correlation graph of neat SLR and SLR/Ag-TNP were displayed in Figure 6d. The resulting graph showed that, as the loading % *w*/*w* of Ag-TNP increases, the flexural strength and flexural modulus increase progressively until 1.0% *w*/*w*, although the sudden decrement was noted when the loading content increased beyond the 1.0% *w*/*w* concentration of Ag-TNP (refer to redline in Figure 6d). The flexural strength and flexural modulus of the 3D printed samples of neat SLR were observed as the lowest compared to all the SLR/Ag-TNP nanocomposites. Overall, the sample SLR/Ag-TNP-1.0 shows the adequate flexural strength and flexural modulus among all the other samples with an increment of 71.8% and 59%, respectively, compared to that of neat 3D printed SLR. The elevated loading of Ag-TNP (higher than 1.0% *w*/*w*) would pile into the SLR system, which caused the lower cross-linking density existing in SLA nanocomposites, directing to a weakening of mechanical behaviors of SLR/Ag-TNP nanocomposites, which results in the diminution of flexural and tensile properties [70]. In order to understand the mechanical properties enhancement in a better way, the flexural strength, tensile strength, % elongation at break, and Young’s modulus of the neat SLR and SLR/Ag-TNP nanocomposites were endowed with Table 1 and Table 2, subsequent to the effects with Figure 6c,d, respectively.

**Figure 7:**
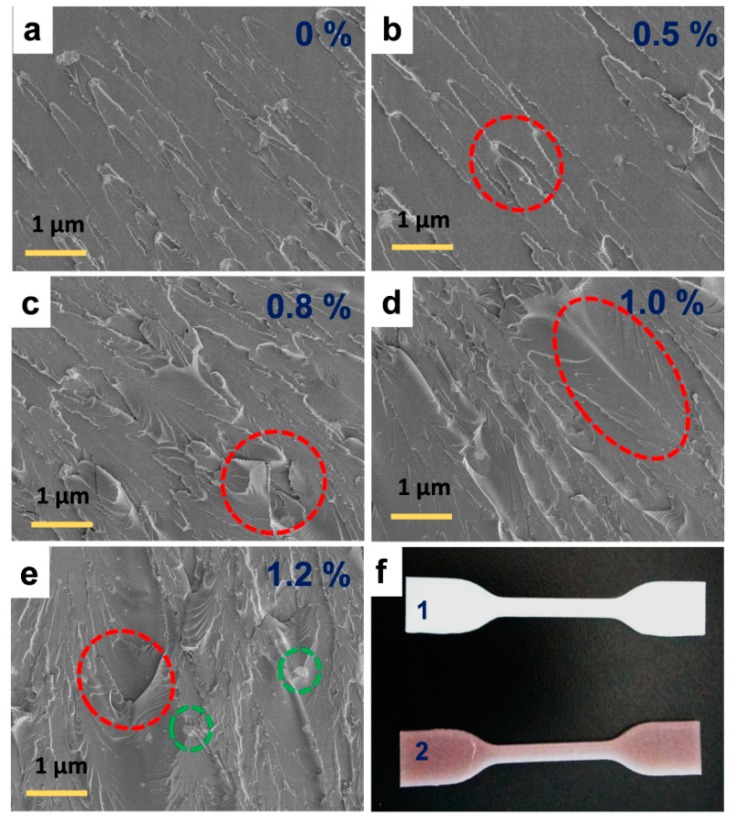
FESEM fracture surface analysis of tensile tested dog-bone specimens of SLA 3D printed samples: (**a**) neat SLR (smooth and less fissure surface), (**b**) SLR/Ag-TNP-0.5, (**c**) SLR/Ag-TNP-0.8 (the marked redline circles represents the uneven cracks), (**d**) SLR/Ag-TNP-1.0 (the highlighted redline demonstrates the uniform dispersion and high rough surface), (**e**) SLR/Ag-TNP-1.2 (the highlighted redline circles shows high roughness and the marked green area representing the possible agglomeration of incorporated Ag-TNP nanoparticles), (**f**) the digital photography of 3D printed dog-bone shaped SLA model samples for tensile strength before analysis (**1**) neat SLR and (**2**) SLR/Ag-TNP-1.0 sample.

For the better perception of the mechanical improvement over the effect of Ag-TNP on the SLR/Ag-TNP nanocomposites, the crack surfaces of the tested tensile trials of neat SLR and SLR/Ag-TNP were investigated by the assistance of FESEM analysis, and the observed morphology images were presented with the Figure 7. The fracture surfaces of neat SLR (refer to Figure 7a) were noted as moderately even and very smooth surfaces had few linear fissures, whereas the fractured surfaces of the SLR/Ag-TNP nanocomposites (refer to Figure 7b–e) were observed with uneven and rough surfaces having some tears without linear cracks. Performing as stress concentrators, the incorporated subsistence of Ag-TNP may power the tear to proliferate along a convoluted path and promote the configuration of numerous micro-fissures. However, when the loading of Ag-TNP increases to 1.2% *w*/*w*, the agglomeration of Ag-TNP was noticed remarkably, which may affect the curing followed by a cross-linking process of the SLR and persuade the development of micro-breakdowns, ensuing with a worsening of mechanical behaviors, together with a considerable lessening in tensile properties (refer to Figure 6c and Table 1) and flexural properties (refer to Figure 6d and Table 2).

**Figure 8:**
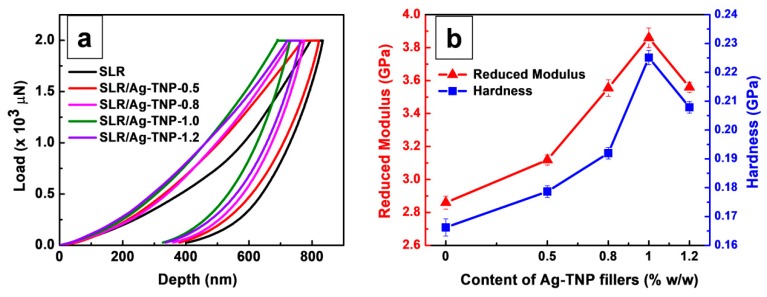
The nanoindentation analysis of neat SLR and SLR/Ag-TNP nanocomposites (**a**) the indentation load–depth curves for neat SLR and SLR/Ag-TNP nanocomposites with respect to different concentrations, (**b**) the observed variation of hardness (blue patterns) and reduced modulus (red patterns) of the SLR/Ag-TNP nanocomposites, where Ag-TNP content used as in % *w*/*w* of 0, 0.5, 0.8, 1.0, and 1.2.

The nanoindentation analysis was demonstrated to examine the micro-mechanical behaviors of the 3D printed samples of neat SLR and SLR/Ag-TNP nanocomposites on a small scale, for instance, hardness and reduced modulus. Figure 8a represents a plot of the applied load adjacent to the indentation intensity for the SLR/Ag-TNP and neat SLR nanocomposites. In conformity with Figure 8a, expressing that all the SLR/Ag-TNP nanocomposites’ indentation depths were recorded as reduced spectacularly compared to the neat SLR nanocomposites. The indentation depths for the SLR/Ag-TNP nanocomposites declined as the addition of Ag-TNP increased with the loading of Ag-TNP up to 1.0% *w*/*w*; later, the privileged indentation depth was observed when the loading goes beyond 1.0% *w*/*w* of Ag-TNP, which determines that the enhancement of micro-mechanical behavior for the Ag-TNP incorporated SLR nanocomposites was active at the lowest loading content of Ag-TNP. The privileged indentation depth of beyond 1.0% *w*/*w* loaded Ag-TNP may be the inhibition of the plastic deformation rising because of the agglomeration of nanofillers within the SLR nanocomposites. Figure 8b demonstrates the reduced modulus, and hardness of the 3D printed samples of SLR/Ag-TNP and pure SLR nanocomposites concerning the different loading percentages of Ag-TNP to the SLR nanocomposites. As the increasing incorporation of Ag-TNP varies from 0.5 to 1.0% *w*/*w*, the hardness and the reduced modulus were monitored as remarkable increases. By the addition of 1.0% *w*/*w* of Ag-TNP into the SLR matrix, the hardness (0.225 GPa) and reduced modulus (3.86 GPa) attained the optimized standards and increased by 40.6% and 34.9%, equating to the neat SLR (0.16 GPa for hardness and 2.86 GPa for reduced modulus), respectively. However, through promoting the increasing of Ag-TNP afar 1.0% *w*/*w*, the hardness and reduced modulus values decrease due to the agglomeration of elevated content of Ag-TNP, which leads to the weak interaction between the SLR matrix and Ag-TNP. The experimental results of indentation depth, hardness, and reduced modulus of the SLR/Ag-TNP and pure SLR nanocomposites were supplied with Table 3.

**Figure 9:**
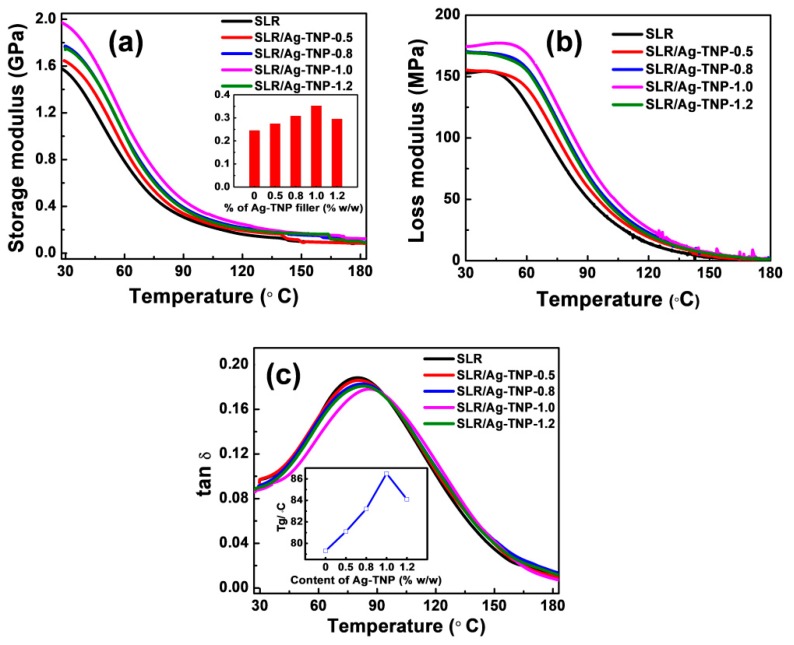
Thermo-mechanical analyses of neat SLR and SLR/Ag-TNP nanocomposites (**a**) comparative storage modulus, (**b**) comparative loss modulus curves, and (**c**) comparative tan *δ* curves. The inserts in (**a**) and (**c**) express the dissimilarity of tan *δ* and storage modulus of SLR/Ag-TNP nanocomposites with different loadings of Ag-TNP in the photo resin, respectively.

The thermo-mechanical behaviors of the SLR/Ag-TNP and neat SLR nanocomposites were investigated with the help of Q800 TA instruments, and the observed results were discussed in this subsection. The DMA method was founded on an easy principle; when a model is subjected to a sinusoidal oscillating stress, its reply is a sinusoidal oscillation with parallel frequency furnishing the material staying within its elastic limits. When the material reacts to the employed oscillating stress entirely elastically, the responding strain wave is in-phase (elastic response or storage), while a viscous material responds with an out-of-phase strain wave (a viscous response or loss). Figure 9a,b represent the deviations of storage modulus (E′) and tan *δ* as a function of temperature, respectively. In accordance with Figure 9a, the storage modulus of 3D printed samples decreased slowly when temperature increased from 25 °C to 180 °C. The storage modulus of the SLR/Ag-TNP nanocomposites was observed as increases with increasing Ag-TNP content with the SLR up to 1.0% *w*/*w*. The increase in the storage modulus signifies the superior scattering and excellent interfacial interaction between the Ag-TNP and SLR matrix in the SLR/Ag-TNP nanocomposites [71]. In addition, Figure 9b demonstrates that, with the increasing nanofillers incorporation, the loss modulus increases gradually, with respect to the storage modulus and glass transition temperature. Later, a sudden decrement was noted when the nanofillers loading reaches over 1 wt%. The loss modulus and storage modulus of SLR/Ag-TNP nanocomposites were decreased when the incorporation of Ag-TNP concentration was more than 1.0% *w*/*w*, due to the elevated nanofillers loading causing reasonable agglomeration of Ag-TNP within the SLR matrix. The sample SLR/AgTNP-1.0 sustains the best storage modulus compared to all other samples with the highest value of 1953.1MPa, compared to a neat SLR value of 1495.2MPa, which was about 30.5% higher than the neat SLR. It ensured that the adding of Ag-TNP with SLR could enhance the storage modulus of 3D printed structure compared to neat SLR.

In addition to the storage modulus, Figure 9c shows the comparative study of tan *δ* curves for the SLR/Ag-TNP and neat SLR nanocomposites. It was investigated from the tan *δ* curves that the glass transition temperature (T*_g_*) of the SLR/Ag-TNP-1.0 (86.5 °C) was the highest and could be improved by an elevated difference of 7.2 °C compared to neat SLR (79.3 °C) (refer to the inset in Figure 9c). Beyond the 1.0 wt% loading concentration, the T*_g_* value decreases due to higher agglomeration leading to the poor dispersion of Ag-TNP within the SLR matrix. The improved T*_g_* could be interpreted as it ensures that the SLR/Ag-TNP-1.0 nanocomposites were thermally highly stable compared to all other samples. The optimized loading content of Ag-TNP expresses a good dispersion within the SLR matrix, and superior cross-linking density directs to a more significant interruption from polymer chain association so that the glass T*_g_* progress was noted at upper temperatures for SLR/Ag-TNP-1.0 nanocomposites. The detailed observed values of DMA analysis were given with Table 4.

#### 3.2.2. Thermal Properties

**Figure 10:**
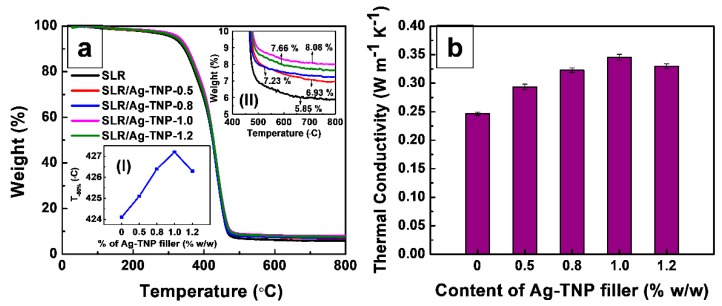
Thermal properties analysis of neat SLR and SLR/nanofillers nanocomposites. (**a**) comparative thermogravimetric analysis (TGA) curves of neat SLR and SLR/nanofillers nanocomposites with different concentrations, and (**b**) comparative thermal conductivity analysis of neat SLR and SLR/nanofillers nanocomposites with respect to various concentrations. The insert graphs of (**I**) and (**II**) in (**a**) illustrates the residual char wt% and variation of T_-50%_ as a function of different % *w*/*w* of Ag-TNP, respectively.

The thermogravimetric analysis of neat SLR and SLR/Ag-TNP nanocomposites was analyzed, and the resulted graph, as shown in Figure 10a. In conformity with Figure 12a and Table 5, in which the residual weight of pristine SLR was noted as the lowest among all the TGA tested samples, and an increasing trend of residual char was noted with the loading percentage of Ag-TNP into the SLR matrix. The residual char weight % in the SLR/Ag-TNP nanocomposites was considerably high compared to pure SLR (5.86), which was showing that the relatively high thermal stability of the SLR/Ag-TNP nanocomposites at high temperatures (for the SLR/Ag-TNP-1.0 sample, the residue weight % was noted as 8.01). However, the loading concentration of Ag-TNP increases beyond the level of 1.0% *w*/*w*, and the sudden decrement of residual char value was noted as a reason for these phenomena was maybe the higher concentration of Ag-TNP could make possible agglomeration within the SLR matrix, which leads to poor dispersion and weakening the thermal stability of SLR/Ag-TNP nanocomposites. The above-mentioned interpretation proposes that the exceptional thermal stability with a lower decomposition rate has been found in the sample SLR/Ag-TNP-1.0. In addition, the value of the mean temperature of the squalor (T_-50%_, temperature) and the residue char value at 800 °C were abridged in Table 5. For the neat SLR nanocomposites, the T_-50%_ was noted as 424.1, and it was significantly increased for the samples of SLR/Ag-TNP nanocomposites. The enhancement of thermal stability can be reorganized by taking into account of the tears, movement, and thermal disintegration of the polymer manacles. With the strong interactions between the incorporated Ag-TNP and SLR matrix, the movements of the polymer chains were partial for the period of the thermal degradation process, which was significantly responsible for the progress in thermal enhancement [72].

Figure 10b represents the thermal conductivity measurements of the neat SLR and SLR/Ag-TNP nanocomposites. From Figure 10b and Table 5, it was investigated that the thermal conductivity of the SLR/Ag-TNP nanocomposites considerably increases when the loading percentage of Ag-TNP increases with the SLR matrix. The average thermal conductivity value of the neat SLR was noted as 0.2465 (W·m^−1^·K^−1^) and the thermal conductivity of the SLR/Ag-TNP nanocomposites increasing with respect to the increases in the % *w*/*w* of Ag-TNP, as it reaches 1.0% *w*/*w* of Ag-TNP nanoparticles with a value of 0.3456 (W·m^−1^·K^−1^) (40.2% of increment in the thermal conductivity more than the neat SLR). When increasing the loading percentage of Ag-TNP in excess of 1.0% *w*/*w*, within the SLR matrix, the deprived thermal conductivity was noted. The thermal conductivity initially increased and was a decreasing trend with the incorporation of Ag-TNP, which was due to the elevated loading of nanofillers possibly causing agglomeration, which leads to poor dispersion within the polymer matrix and lessens the thermal conductivity. Moreover, silver was considered as one of the excellent thermal conductors among the metals. Here, the enhancement of thermal conductivity may be due to the silver decorated TNP attaining very good dispersion within the SLR matrix at lower loading concentration, which could possibly enhance the thermal conductivity of SLR/Ag-TNP nanocomposites [73]. To study the thermal conductivity significance of our research work, the observed thermal conductivity values were compared with other reported photocurable resin/nanocomposites thermal conductivity values. The comparative thermal conductivity was displayed in Table 6.

**Figure 11:**
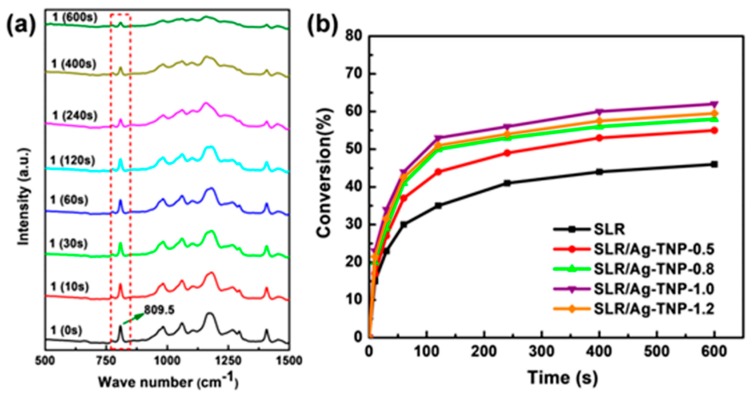
(**a**) a real-time FTIR analysis of photopolymerization of SLR/Ag-TNP-1 nanocomposite in different periods of time intervals starting from 0 s to 600 s under the UV irradiation power of 32 mW/cm^2^, (**b**) percentage conversion graph of neat SLR and SLR/Ag-TNP nanocomposites.

The kinetic study of photo-polymerization reaction of both neat SLR resin and Ag-TNP nanofillers incorporated SLR nanocomposites analyzed with the assistance of Real-Time Fourier transform infrared spectra (RT-FTIR), and the observed results are presented in Figure 11. The photopolymerization kinetics was resolved by the decline of absorption peak intensity around 809–811 cm^−1^ from the FTIR analysis, which was attributed to the twisting vibration mode of double-bond in an acrylic group [79]. The degree of conversion (DC) of a double-bond was calculated from Equation (3) [80,81]:

(3) $$\%\;\mathrm{Conversion}=\frac{A_{0}-A_{t}}{A_{0}}\times 100\%$$

where A_0_ is the initial peak area before the irradiation at 809.5 cm^−1^, while A_t_ is the peak area at irradiation time t at 809.5 cm^−1^ during the polymerization, respectively.

The analysis was executed on the basis of different time intervals to comprehend the photopolymerization progression. Initially, the uncured vat slurry was examined by FTIR, followed by UV curing with an irradiation power of 32 mW/cm^2^ at different time intervals such as 10 s, 30 s, 60 s, 120 s, 240 s, 400 s, and 600 s. The neat SLR and SLR/Ag-TNP-1 slurry analysis is presented in Figure 11a, and all other SLR/nanofillers analyses such as SLR/Ag-TNP-0.5, SLR/Ag-TNP-0.8, and SLR/Ag-TNP-1.2 are displayed in Supplementary Figure S5. The analysis was employed to determine the absorbance intensity of the peak at 809.5 cm^−1^, which is related to acrylate double bond. From the FTIR analysis, the percentage conversion versus time curves was plotted to understand the photopolymerization reactions based on the double bond (C=C) conversion ratio taking place in different time periods. Figure 11b shows that the conversion rates of the photopolymerization reactions were enhanced by the addition of nanofillers content. Figure 11b expresses that the SLR/Ag-TNP-1 nanocomposite showed a better C=C conversion rate of photopolymerization among all other SLR/nanofillers’ composites. The percentage conversion of SLR/Ag-TNP-1 was reached to 62.2% at the 600 seconds, where percent conversion of SLR was only 45.8%. In addition, to investigate the effect of radiation power on the photopolymerization kinetics, as prepared SLR/Ag-TNP-1% nanocomposites have been taken to perform under the different UV power intensities. The recorded RT-FTIR polymerization profiles of the sample with different power densities of UV-irradiation at different time intervals are illustrated in the supplementary Figure S6. The analysis was executed on the basis of different time intervals to realize the photopolymerization evolution. Primarily, the uncured SLR/Ag-TNP-1 slurry was examined by FTIR, followed by UV curing with an irradiation power of 24 mW/cm^2^ at different time intervals such as 10 s, 30 s, 60 s, 120 s, 240 s, 400 s, and 600 s. The same procedure carried out for SLR/Ag-TNP-1 with different power densities of UV irradiation of 28 mW/cm^2^ and 32 mW/cm^2^ in the above-mentioned time intervals. The percentage conversions of SLR/Ag-TNP-1 at 600s in the different UV exposure power densities of 24 mW/cm^2^, 28 mW/cm^2^ and 32 mW/cm^2^ are 55.8%, 59.1%, and 62.2%, respectively.

### 3.3. Thermal and Mechanical Reinforcement Mechanism of SLR/Ag-TNP 3D Printed Samples

**Figure 12:**
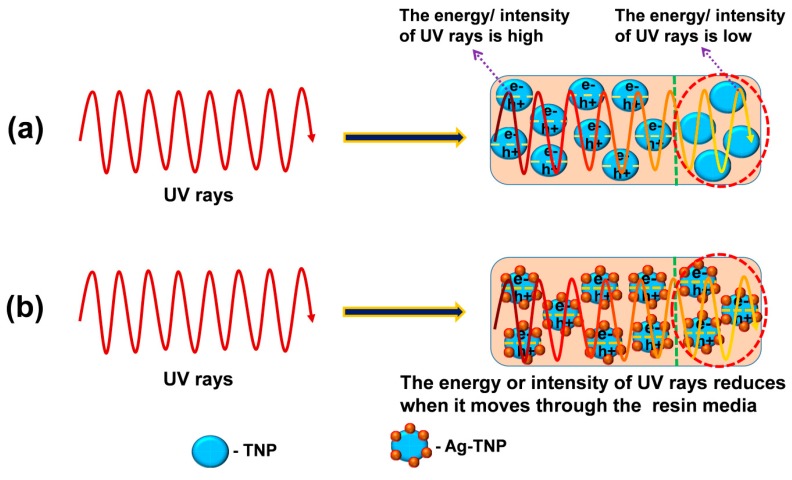
The proposed induced photopolymerization mechanism of SLR/nanofillers schematic illustration of UV light penetration through the (**a**) SLR/high energy bandgap semiconducting NPs (e.g., TiO_2_) (the dotted red circles represents there is no possible electron hole pairs generation when the UV intensity decreases), and (**b**) SLR/low energy bandgap semiconducting NPs (e.g., Ag-TNP) (the dotted red highlights demonstrates that, the successful electron–hole pairs formation even at lower intensity of UV lights). The energy/intensity of UV rays reduces (the red color lines indicate higher intensity and pale yellow color lines indicates lower intensity) when it undergoes deep penetration through the resin composites.

Figure 12 demonstrates that the energy or intensity of UV light decreases with respect to its penetration depth increasing through the photo resin nanocomposites. The incorporated low bandgap semiconducting nanoparticles (here, Ag-TNP) can able to generate more electron–hole pairs compared to high bandgap semiconducting NPs (here, TiO_2_) at deep penetration depth, where the energy of UV light was less. For the better understandings, we have performed the photopolymerization kinetic study of SLR/TNP-1 and SLR/Ag-TNP-1 nanocomposites via real-time FTIR study and the % conversion of C=C was calculated from the analysis; the resulting graphs were displayed in the supplementary Figure S7. Thus, Ag-TNP can induce the photopolymerization via fast and strong cross-linking with the SLA resin matrix, which leads to better improvement over mechanical and thermal properties of the SLR/nanofillers 3D printed polymer nanocomposites. The semiconducting nanoparticles can able to induce photopolymerization by the process of possible creation of electron hole pair in the presence of UV light, which leads to enhancing the thermal and mechanical properties of a polymer matrix [82,83].

The UV rays on the surface of semiconducting Ag-TNP can experience a photo-excitation which causes the multiplication of electrons in conduction band (CB) and holes in valence band (VB) as presented in Equation (4) [84]:

Ag-TNP + hν → Ag-TNP (e^−^_CB_ + h^+^_VB_). (4)

The as-developed holes in VB can oxidize acrylic monomers extending to the production of free radicals (refer to the Equation (5)), which can encourage the initiation for polymerization chemical reaction (refer to the Equation (6)):

h^+^_VB_ + MA → M A^•^+ H^+^ (5)

M A^•^ + MA → PR (6)

In addition, the photo-reduction reaction by CB electrons experiences the reaction correspondingly:

e^−^_CB_ + MA → MA^•−^ (7)

MA^•−^ + H^+^ → H MA^•^ (8)

The recipe of electrons in CB with protons induces the establishment of atomic hydrogen compiled by H MA^•^ radicals, which aids with starting the polymerization process:

e^−^_CB_ + H^+^ → H^•^ (9)

MA + H^•^ → H MA^•^ (10)

where, MA, MA^•^, and PR symbolizes, the acrylate based monomer, monomer radical and polymer, respectively.

With regard to the semiconducting behaviour of the incorporated metal–metal oxide nanofillers (Ag-TNP), the photo-absorption process of Ag-TNP hybrids is able to generate valence band holes and conduction band electrons on the surface of Ag-TNP upon UV irradiation [52]. However, the electrons and holes generated from the Ag-TNP nanoparticles assistance with the formation of monomer radicals, which can react further with the monomer and thereby initiate polymerization chains, but the propagation of polymer chain occurs due to the monomer radicals. Hence, this polymer reaction was considered as free radical photopolymerization [85]. Herein, the photopolymerization can occur in three routes. Firstly, holes, generated in a valence band can oxidize organic molecules such as acrylic monomer leading to the formation of monomer radical (refer to Equation (5)), which can react further with the monomer and thereby initiate polymerization chains (shown in Equation (6)). Secondly, photoreduction by conduction band electrons can take place at the same time and form a negative charged monomer radical (Equation (7) and Equation (8)). The proton created as a by-product from the first route, which can react with the negative charged monomer radical, can form H MA^•^ radicals (Equation (8)), which can react further with the monomer to form the polymer chains. Thirdly, the interaction of conduction band electrons with protons occurs, leading to atomic hydrogen formation (Equation (9)). The latter attack to the monomer can form HM radicals (Equation (10)) capable of initiating polymerization of the acrylic monomer [86].

It was affirmed by the mechanical and thermal analysis of Ag-TNP incorporated 3D printed samples, in which the sample SLR/Ag-TNP-1.0 exhibited a prominent improvement of thermal and mechanical properties. The splendid property enrichment was accomplished for SLR/Ag-TNP-1.0, entailing that the introduced Ag-TNP-1.0 having an extremely crystalline anatase phase and the scummiest energy bandgap can be proficient to engender a quick massive figure of electron–hole pairs, which leads to the burly cross-linking among monomers and oligomers in SLR/Ag-TNP nanocomposites.

## 4. Conclusions

The semiconducting low energy bandgap Ag-TNP was synthesized through the sol-gel method and utilized as nanofillers in the stereolithography resin matrix to enhance the thermal and mechanical properties of 3D printing samples. The research study shows that the introduction of Ag-TNP in the SLR matrix can extensively enhance the mechanical and thermal behaviors of 3D printed samples. The mechanical properties of the 3D printed samples were increasing remarkably when the Ag-TNP was added into the SLR up to 1.0% *w*/*w* and decreased when the addition went beyond 1.0% *w*/*w* due to the agglomeration of higher concentration of nanoparticles causing the poor dispersion and improper cross-linking density. In addition, the thermal conductivity and thermal stability of the 3D printed samples of SLR/Ag-TNP nanocomposites were increased with the increasing loading percentage of Ag-TNP with the SLR matrix. This study presents a promising result and gives an instructive role to manufacture of stereolithography 3D printing parts by using Ag-TNP dispersed in the SLR matrix with excellent performance in the mechanical and thermal properties, which accelerates the expansion and sensible function of SLR/Ag-TNP nanocomposites and widens the applications of additive manufacturing technology.

## Figures and Tables

**Table 1:** The tensile strength and tensile modulus of SLR/Ag-TNP and neat SLR nanocomposites.

Sample	Tensile Properties		Strain (%)
	Strength (MPa)	Modulus (GPa)	
**Neat SLR**	27.8 ± 1.3	1.5 ± 0.2	4.9 ± 0.5
**SLR/Ag-TNP-0.5**	33.2 ± 0.8	1.8 ± 0.1	4.5 ± 0.3
**SLR/Ag-TNP-0.8**	39.7 ± 1.1	2.0 ± 0.1	4.0 ± 0.2
**SLR/Ag-TNP-1.0**	44.7 ± 0.9	2.3 ± 0.1	3.6 ± 0.4
**SLR/Ag-TNP-1.2**	43.5 ± 0.8	2.1 ± 0.1	2.9 ± 0.3

SLR: Stereolithography resin; Ag-TNP: Ag decorated TiO_2_ nanoparticles

**Table 2:** Results of flexural strength and flexural modulus of SLR/Ag-TNP and neat SLR nanocomposites.

Sample	Flexural Properties		Strain (%)
	Strength (MPa)	Modulus (GPa)	
**Neat SLR**	42.0 ± 1.2	2.2 ± 0.10	5.2 ± 0.3
**SLR/Ag-TNP-0.5**	51.5 ± 1.5	2.6 ± 0.05	4.8 ± 0.2
**SLR/Ag-TNP-0.8**	62.1 ± 1.1	2.9 ± 0.10	4.3 ± 0.4
**SLR/Ag-TNP-1.0**	70.7 ± 2.1	3.5 ± 0.10	4.0 ± 0.4
**SLR/Ag-TNP-1.2**	64.7 ± 1.9	3.1 ± 0.05	3.1 ± 0.5

**Table 3:** Extracted results of pure SLR and SLR/AgTiO_2_ nanocomposites from the nanoindentation analysis.

Sample	Indentation Depth (nm)	Reduced Modulus (GPa)	Hardness (GPa)
Neat SLR	834.07	2.859 ± 0.03	0.1662 ± 0.0030
SLR/Ag-TNP-0.5	822.16	3.118 ± 0.05	0.1786 ± 0.0020
SLR/Ag-TNP-0.8	774.37	3.554 ± 0.04	0.1919 ± 0.0020
SLR/Ag-TNP-1.0	728.52	3.859 ± 0.06	0.2251 ± 0.0025
SLR/Ag-TNP-1.2	761.06	3.559 ± 0.03	0.2078 ± 0.0020

**Table 4:** The storage modulus and tan *δ* values of neat SLR and SLR/Ag-TNP nanocomposites.

Sample	Storage Modulus (MPa)		tan *δ* Peak Height	T*_g_* (°C)
	30 °C	100 °C		
Neat SLR	1495.2	245.1	0.188	79.3
SLR/Ag-TNP-0.5	1637.4	264.8	0.185	81.1
SLR/Ag-TNP-0.8	1769.9	308.9	0.182	83.2
SLR/Ag-TNP-1.0	1953.1	350.5	0.177	86.5
SLR/Ag-TNP-1.2	1751.1	295.5	0.179	84.1

**Table 5:** Results of TGA and Thermal conductivity of SLR/Ag-TNP and neat SLR nanocomposites.

Sample	T_-50%_ (°C)	Residual Char (wt%)	Thermal Conductivity (W·m^−1^·K^−1^)
Neat SLR	424.1	5.86	0.2465 ± 0.003
SLR/Ag-TNP-0.5	425.1	6.93	0.2934 ± 0.005
SLR/Ag-TNP-0.8	426.4	7.24	0.3228 ± 0.004
SLR/Ag-TNP-1.0	427.2	8.01	0.3456 ± 0.005
SLR/Ag-TNP-1.2	426.3	7.63	0.3298 ± 0.004

**Table 6:** The photocurable resin/nanofillers thermal conductivity comparison study with different nanocomposites.

Nanocomposites	Thermal Conductivity (W·m^−1^·K^−1^)	References
SLR/Ag-TNP-1.0	0.34	This work
^®^ SLR/ANT800	0.29	[74]
^@^ MPTS/hBN-5%	0.30	[75]
^#^ SU-8/FGS-2%	0.36	[76]
^$^ CE/BNNT-1.5%	0.40	[77]
^&^ UV curable polyacrylate/PGN15	0.29	[78]
* UV curable polyacrylate/PGN25	0.27	[78]

^®^ Stereolithography resin incorporated with 800 °C annealed anataseTiO_2_ nanoparticles. ^@^ Photocurable acrylic-based photopolymer composites with 5 wt% of hexagonal boron nitride (hBN) modified with γ-Methacryloxypropyltrimethoxysilane. ^#^ Photocurable SU-8 epoxy-based composites containing 2 wt% functionalized graphene sheets (FGS). ^$^ Photo curable epoxy-based composites which contain (3,4-Epoxy cyclohexylmethyl-3,4-epoxycyclohexane carboxylate (CE) with 1.5 wt% of Boron Nitride nanotubes (BNNTs). ^&^ UV-curable polyacrylate incorporated with 15 nm size gold nanocomposites (PGN15). * UV-curable polyacrylate incorporated with 25 nm size gold nanocomposites (PGN25).

## Supplementary Materials

The following are available online at https://www.mdpi.com/2079-4991/10/2/217/s1, Figure S1: A detailed schematic illustrates of synthesis route of Ag-TNP. Figure S2: The step by step detailed preparation route of SLR/Ag-TNP nanocomposites. Figure S3: The digital image of the Dream 3D-C200 SLA 3D printing device, which was utilized for this research work. Figure S4: XPS spectrum analysis of as-prepared TiO_2,_ and Ag/TNP (a) survey spectrum (black spectrum represents TiO_2_ and red spectrum represents Ag-TNP), (b) Deconvoluted XPS spectra of Ti, and (c) Deconvoluted XPS spectra of Ag, (c) Deconvoluted XPS spectra of O. Figure S5: A real-time FTIR analysis of photopolymerization of (a) neat SLR, (b) SLR/Ag-TNP-0.5, (c) SLR/Ag-TNP-0.8, and (d) SLR/Ag-TNP-1.2, nanocomposites in different periods of time intervals starting from 0 s to 600 s under the UV irradiation power of 32 mW/cm^2^. Figure S6: (a) percentage conversion graph of SLR/Ag-TNP-1 nanocomposite in different power densities of UV-irradiation monitored by RT-FTIR. (b), (c), and (d) are real-time FTIR analysis of photopolymerization of SLR/Ag-TNP-1 nanocomposites in different periods of time intervals starting from 0 s to 600 s under UV irradiation power densities of 24 mW/cm^2^, 28 mW/cm^2^ and 32 mW/cm^2^, respectively. Figure S7: (a) real-time FTIR analysis of SLR/TNP-1 nanocomposite and (b) percentage conversion graph of photopolymerization of SLR/TNP-1 nanocomposite (black line) and SLR/Ag-TNP-1 (red line) nanocomposite, under the UV irradiation power of 32 mW/cm^2^ in different periods of time intervals starting from 0 s to 600 s. Table S1: The composition of mixture used for SLA photoresin preparation.
