# Supplementary material for: A Novel Approach to Enhance Mechanical and Thermal Properties of SLA 3D Printed Structure by Incorporation of Metal–Metal Oxide Nanoparticles
Source: Nanomaterials (Basel). 2020 Jan 27;10(2):217. doi: 10.3390/nano10020217 (PMC7074857; doi:10.3390/nano10020217)
Supplement: Supplementary file 1 [file nanomaterials-10-00217-s001.pdf]

A novel approach to enhance mechanical and thermal properties of SLA 3D printed structure by incorporation of metal-metal oxide nanoparticles

Suhail Mubarak [1, 2], Duraisami Dhamodharan [1, 2], Manoj B. Kale [1, 2], Nidhin Divakaran [1, 2], T. Senthil [3], Sathiyanathan. P [1], Lixin Wu *, [1,4], Jianlei Wang*, [1]

[1] CAS Key Laboratory of Design and Assembly of Functional Nanostructure, and Fujian Key Laboratory of Nanomaterials, Fujian Institute of Research on the Structure of Matter, Chinese Academy of Sciences, Fuzhou, Fujian 350002, P. R. China

[2] University of Chinese Academy of Sciences, Beijing 100049, P. R. China

[3]Advanced Research School for Technology and Product Simulation, Central Institute of Plastics Engineering and Technology, Chennai 600032, India

[4] National Engineering Research Center for Optoelectronic Crystalline Materials

*Corresponding Authors. E-mail: lxwu@fjirsm.ac.cn (L. Wu), jlwang@fjirsm.ac.cn (J. Wang)

Supplementary Materials

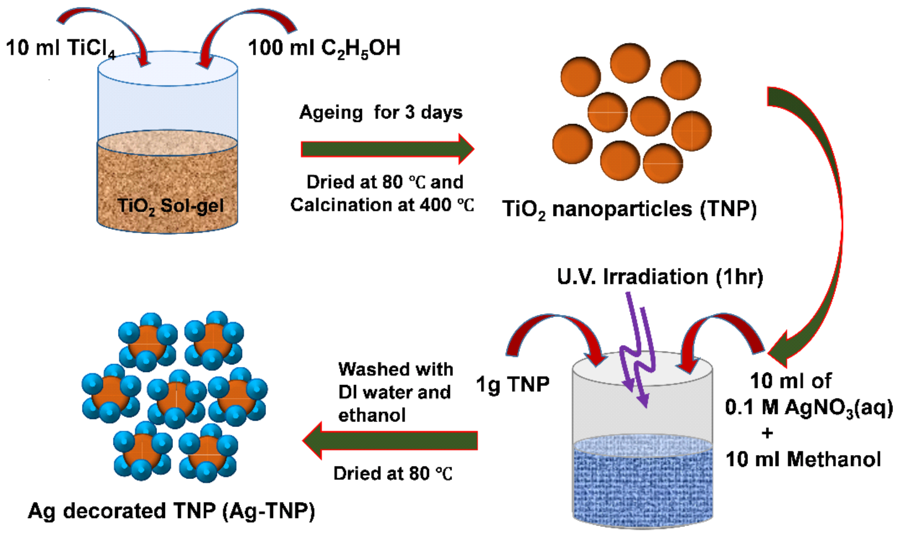

Figure S1 Detailed schematic illustrates of synthesis route of Ag-TNP.

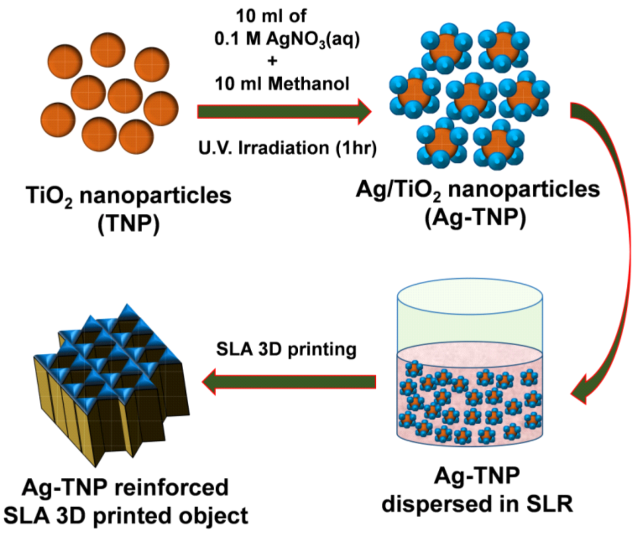

Figure S2 The step by step detailed preparation route of SLR/Ag-TNP nanocomposites.

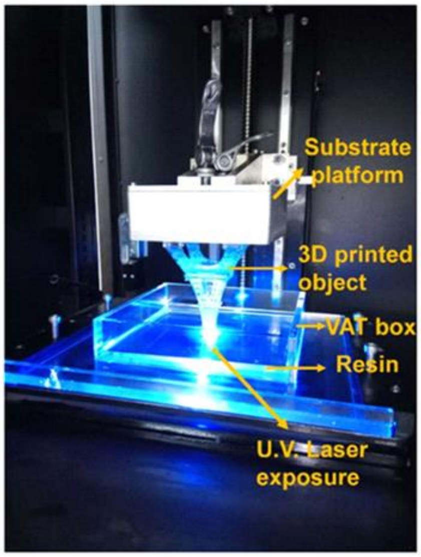

Figure S3 The digital image of the SLA 3D printing device, which was utilized for this research work.

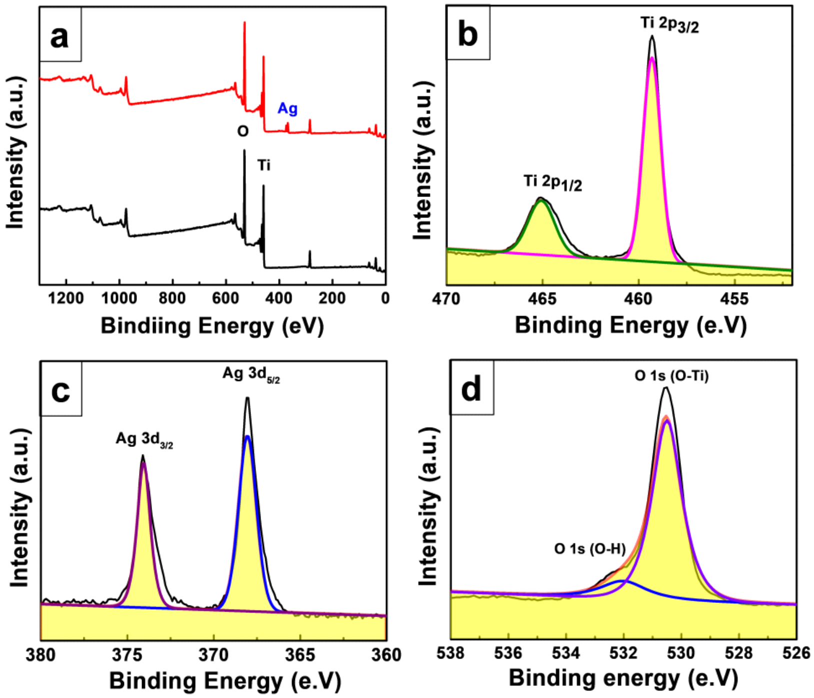

Figure S4 XPS spectrum analysis of as-prepared TiO_2, and Ag/TNP (a) survey spectrum (black spectrum represents TiO_2 and red spectrum represents Ag-TNP), (b) Deconvoluted XPS spectra of Ti, and (c) Deconvoluted XPS spectra of Ag, (c) Deconvoluted XPS spectra of O.

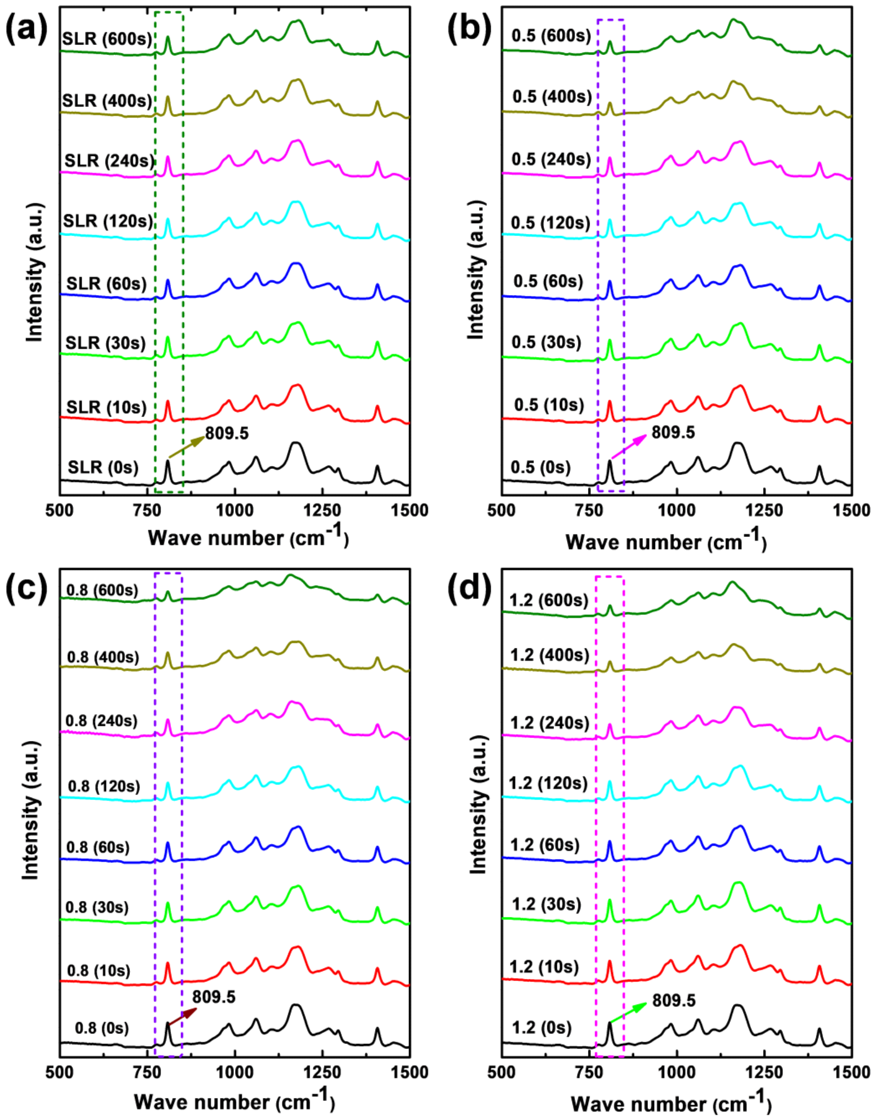

Figure S5 A real time FTIR analysis of photopolymerization of (a) neat SLR, (b) SLR/Ag-TNP-0.5, (c) SLR/Ag-TNP-0.8 and (d) SLR/Ag-TNP-1.2, nanocomposites in different periods of time intervals starting from 0 s to 600 s under the UV irradiation power of 32 mW/cm^2.

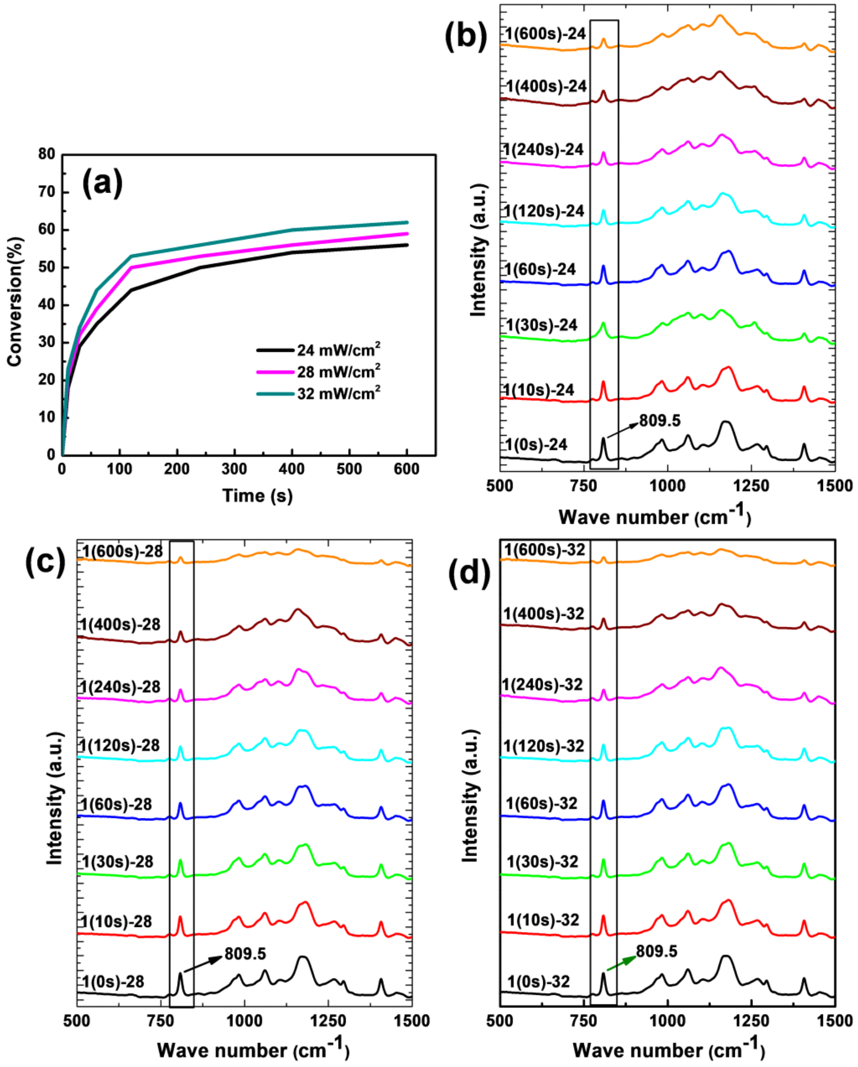

Figure S6 (a) Percentage conversion graph of SLR/Ag-TNP-1 nanocomposite in different power densities of UV-irradiation monitored by RT-FTIR. (b), (c) and (d) are real time FTIR analysis of photopolymerization of SLR/Ag-TNP-1 nanocomposites in different periods of time intervals starting from 0 s to 600 s under UV irradiation power densities of 24mW/cm^2, 28mW/cm^2 and 32mW/cm^2 respectively.

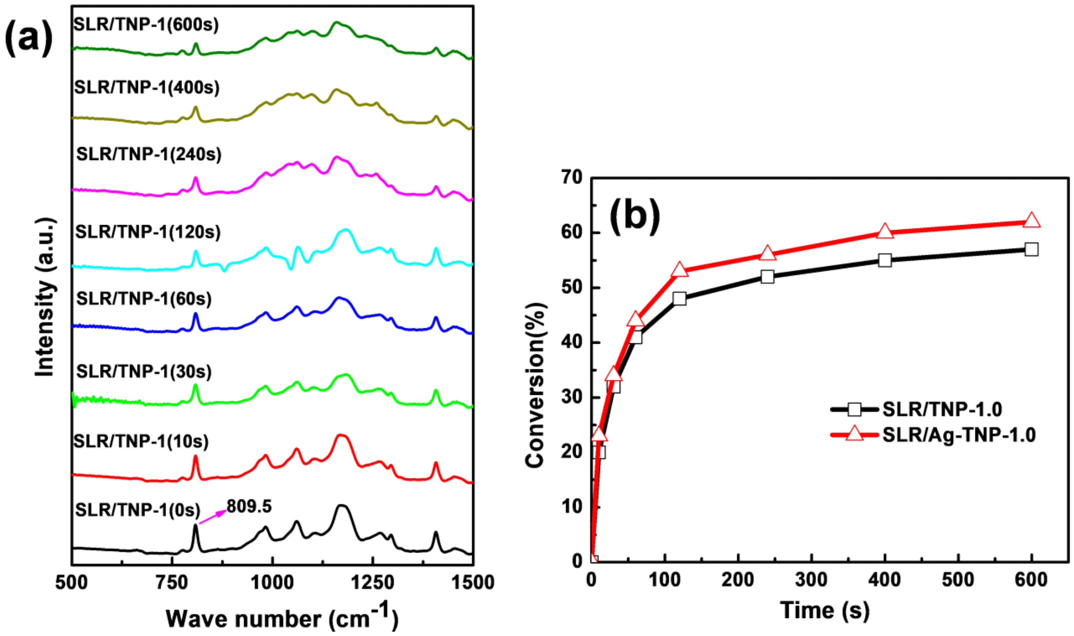

Figure S7 (a) Real time FTIR analysis of SLR/TNP-1 nanocomposite and (b) Percentage conversion graph of photopolymerization of SLR/TNP-1 nanocomposite (black line) and SLR/Ag-TNP-1 (red line) nanocomposite, under the UV irradiation power of 32mW/cm^2 in different periods of time intervals starting from 0 s to 600 s.

Table S1 The composition of mixture used for SLA photoresin preparation.

**Combination of photoresin**	**Mass ratio (% w/w)**
CN9010	33
CN991	32
SR209	20.5
HEMA	6
HPMA	8
TPO	0.5
